# The UKCAT-12 study: educational attainment, aptitude test performance, demographic and socio-economic contextual factors as predictors of first year outcome in a cross-sectional collaborative study of 12 UK medical schools

**DOI:** 10.1186/1741-7015-11-244

**Published:** 2013-11-14

**Authors:** I C McManus, Chris Dewberry, Sandra Nicholson, Jonathan S Dowell

**Affiliations:** 1 UCL Medical School, University College London, Gower Street, London WC1E 6BT, UK; 2 Research Department of Clinical, Educational and Health Psychology, Division of Psychology and Language Sciences, University College London, Gower Street, London WC1E 6BT, UK; 3 Department of Organizational Psychology, Birkbeck, University of London, Malet Street, Bloomsbury, London WC1E 7HX, UK; 4 Institute of Health Science Education, Queen Mary London, Turner Street, London E1 2AD, UK; 5 Undergraduate Medical Education, Ninewells Hospital and Medical School, Dundee, Scotland DD1 9SY, UK

**Keywords:** Medical student selection, Educational attainment, Aptitude tests, UKCAT, Socio-economic factors, Contextual measures

## Abstract

**Background:**

Most UK medical schools use aptitude tests during student selection, but large-scale studies of predictive validity are rare. This study assesses the United Kingdom Clinical Aptitude Test (UKCAT), and its four sub-scales, along with measures of educational attainment, individual and contextual socio-economic background factors, as predictors of performance in the first year of medical school training.

**Methods:**

A prospective study of 4,811 students in 12 UK medical schools taking the UKCAT from 2006 to 2008 as a part of the medical school application, for whom first year medical school examination results were available in 2008 to 2010.

**Results:**

UKCAT scores and educational attainment measures (General Certificate of Education (GCE): A-levels, and so on; or Scottish Qualifications Authority (SQA): Scottish Highers, and so on) were significant predictors of outcome. UKCAT predicted outcome better in female students than male students, and better in mature than non-mature students. Incremental validity of UKCAT taking educational attainment into account was significant, but small. Medical school performance was also affected by sex (male students performing less well), ethnicity (non-White students performing less well), and a contextual measure of secondary schooling, students from secondary schools with greater average attainment at A-level (irrespective of public or private sector) performing less well. Multilevel modeling showed no differences between medical schools in predictive ability of the various measures. UKCAT sub-scales predicted similarly, except that Verbal Reasoning correlated positively with performance on Theory examinations, but negatively with Skills assessments.

**Conclusions:**

This collaborative study in 12 medical schools shows the power of large-scale studies of medical education for answering previously unanswerable but important questions about medical student selection, education and training. UKCAT has predictive validity as a predictor of medical school outcome, particularly in mature applicants to medical school. UKCAT offers small but significant incremental validity which is operationally valuable where medical schools are making selection decisions based on incomplete measures of educational attainment. The study confirms the validity of using all the existing measures of educational attainment in full at the time of selection decision-making. Contextual measures provide little additional predictive value, except that students from high attaining secondary schools perform less well, an effect previously shown for UK universities in general.

## Background

For many years the primary criterion used to select medical school applicants world-wide has been measures of educational attainment. In the UK, General Certificate of Education (GCE) A-levels, for those educated in England, Wales and Northern Ireland, and Scottish Qualifications Authority (SQA) Highers for most of those educated in Scotland, have been the norm. A-levels have been shown to be valid predictors of outcome, both during the medical course [1] and later in medical careers [2], and for university education in general [3], and more generally in Europe, secondary school grades are predictive of university performance [4]. Educational attainment is also important in medical student selection in many countries, such as Australia, Denmark, Iran, The Netherlands [5], New Zealand and Thailand [6]. However, in recent years the continued reliance on such attainment measures as the sole or principal basis for medical student selection has been questioned for three reasons. First, A-levels and Scottish Highers, which are typically taken in the final year of secondary school, have over the past two decades shown continual increases in grades attained (so-called, ‘grade inflation’). The result is that a large proportion of UK applicants to medical schools now achieve the highest grades (a ceiling effect), so that discriminating between them for the purpose of selection is increasingly problematic. Second, selection on the basis of A-level and Scottish Higher grades may be construed as unfair, because students who have attended selective schools, including independent secondary schools charging high fees, tend to obtain higher grades than others, although this in part may reflect different intake profiles. Third, there are concerns that academic assessment alone may not necessarily select the candidates that possess the behavioral or non-cognitive attributes thought desirable in medical students and doctors. As a consequence, able but economically or socially disadvantaged children attending comprehensive secondary schools might be less likely to obtain a place at medical school than students of equal ability attending selective or private sector secondary schools. Arising from such concerns has also been a growing awareness of the paucity of large-scale, longitudinal studies which have examined performance in medical school in relation to a wide range of measures collected during selection. Without such studies it is difficult to assess the validity and defensibility of the processes currently used to select medical students.

In response to these challenges, most UK medical schools in recent years have used aptitude tests as a supplementary selection technique. An aptitude test usually consists of a series of multiple choice sub-tests. Sub-tests are developed to assess specific aptitudes thought to be relevant for performance at medical school (for example, numerical ability and verbal ability). Unlike secondary school examinations, which measure attainment in relation to a particular discipline (for example, chemistry), aptitude tests are specifically designed to measure intellectual and behavioral capacity, and the potential to perform well in a particular role. Furthermore, aptitude tests offer considerable operational advantages: they can be taken in standardized settings by anyone, whatever their cultural or educational background, at geographical locations all over the world; they can assess people against items for which the difficulty level has been pre-established; they can be completed in a short time (typically less than three hours); and candidates’ performance on the test can be computed immediately. Although aptitude tests are used in medical selection in many countries including Australia [7], Chile [8], Germany [9,10], Pakistan [11], Italy [12], Mexico [13], Switzerland [14], the United States [15,16] and the UK [17,18], research on the extent to which they successfully predict medical performance is patchy. Given the high-stakes nature of medical selection it is clearly important to establish the predictive validity of aptitude tests, and the extent to which they add value to more traditional approaches to selection, such as previous educational attainment, using datasets large enough to provide substantial statistical power. Here we report the results of the first large-scale analysis of the predictive and incremental validity of UK Clinical Aptitude Test (UKCAT), the aptitude test used in the selection of medical students in most UK medical schools.

Aptitude tests can be used as a stand-alone selection device or, more typically, to supplement the existing information on which selection decisions are made, such as a candidate’s secondary school attainment, Universities and Colleges Admissions Service (UCAS) personal statements and medical school interview performance. Because these tests can be specifically designed to differentiate between highly able candidates, and to do so in relation to the particular characteristics required in the medical profession, they can potentially discriminate appropriately between those with equally good attainment at A Level and Scottish Highers. In addition, by measuring the extent to which candidates’ possess aptitudes relevant to the medical profession, rather than their level of school-related educational attainment, aptitude tests may help to widen participation in medicine [19].

The two main aptitude tests currently in operation in the United Kingdom are the UK Clinical Aptitude Test (UKCAT) [20], which is the principle interest of the present study, and the Biomedical Admissions Test (BMAT) [21,22]. For admission in 2013, UKCAT is being used by 26 UK medical schools, and BMAT by 4 UK medical schools. If aptitude tests are to be of added value in addressing the problem of ceiling effects in educational qualifications, they must predict the future performance of medical school candidates over and above that provided by A-levels and Scottish Highers [23]. In addition, if they are to widen participation in the medical profession, it is desirable that scores on UKCAT and BMAT should be less strongly associated with selective secondary schooling than A levels or Scottish Highers.

At present, both UKCAT and BMAT are somewhat controversial [23,24], mainly because of concerns about how well they predict performance at medical school. However, there is also concern over the cost deterring poorer applicants and the effects of coaching [25], which in general can have an effect size of about .26 [26]. To date there have been four studies of the predictive ability of UKCAT [17,27-29], all of which are moderately small (Ns = 292, 307, 204 and 146), and have widely varying conclusions, from a study suggesting the test provides no significant prediction [30] to claims of significant predictive ability [17]. Additionally, one study [31] found no relationship between UKCAT scores and scores on admissions interviews. An important consideration in determining the validity of any aptitude test is that as well as demonstrating predictive validity in its own right, it should also show incremental validity when used with current and accepted methods of selection, which at present for medicine are primarily achievement tests taken in secondary education, coupled in many cases with interviews. That is particularly important as tests such as the American Medical College Admission Test (MCAT), which have both aptitude and attainment components, typically find that most of the prediction is due to the attainment component, rather than the aptitude component [32].

Given the widespread adoption of UKCAT for medical school selection since its introduction in 2006 [33], a more comprehensive examination of the extent to which the test can successfully predict performance and widen participation is required. In this article we address this issue by examining the relationships among multiple predictors of medical school performance (including UKCAT, A levels and Scottish Highers, and a broad range of contextual and socio-cultural measures, including selective schooling), in relation to the first year medical school performance of 4,811 students studying at 12 English and Scottish medical schools in three cohorts who took the UKCAT in 2006 to 2008, entered medical school in 2007 to 2009, and completed their first year in 2008 to 2010.

Although the primary impetus for the present study was to evaluate UKCAT in the context of medical student selection, the UKCAT-12 study can also be used to address a wider set of important issues. As yet there has been no large-scale, prospective study of medical student performance drawing on a wide range of measures which might predict that performance (including detailed socio-economic background measures) across a substantial sample of medical schools. UKCAT-12 provides exactly that, giving not only a platform from which to ask many questions about the nature of medical student selection and education and the assessment of the effects of a large number of different background measures, but also allowing a determination of the extent to which different measures might have different predictive values in different medical schools. Although, therefore, a prime interest of the present study is to evaluate UKCAT, it also represents the first, long-term, large-scale study of medical student training in the UK. Important features of the present analysis are that the sample is large (nearly 5,000 students), it is diverse and representative of a range of medical schools (12 medical schools taking part), it is extended over time (the data being collected across several years), and there is a ‘hard’ outcome measure in the form of medical school examination results on a continuous scale. That means the current study has high statistical power, and also makes it possible to compare medical schools in order to assess the degree to which the conclusions can be generalized across medical schools. Thus, the accumulating database associated with the UKCAT provides an important opportunity not only to assess the effectiveness of the UKCAT, but also to assess the influence of a far broader range of issues concerning how educational, demographic and social factors influence medical school outcome, including those assessed with the ‘contextual measures’ which will soon be available for routine use during selection.

### Aims of the analysis

The present analysis takes into account the aims which UKCAT set for itself [20,34], as well as various previous studies of aptitude tests (and the criticisms of those studies). It therefore looks at:

◦ The predictive validity of A-levels and Scottish Highers for performance in the first year of medical school studies.

◦ The predictive validity of UKCAT for performance in the first year of medical school studies.

◦ The incremental validity of UKCAT over and above existing measures of educational attainment, both General Certificates of Secondary Education (GCSEs)/AS-levels/A-levels and Scottish Highers/Advanced Highers.

◦ The specific predictive ability, with and without taking educational attainment into account, of the four subscales of UKCAT.

◦ An assessment of whether ‘Theory ‘and ‘Skills’ measures at medical school are predicted differently by educational attainment and UKCAT aptitude measures.

◦ An assessment of whether the predictive validity of any of the measures is different in the 12 medical schools that have taken part in the study.

◦ The role of demographic and socio-economic factors in moderating any of the findings.

It should be noted that the present study is restricted to medical school entrants and, therefore, it cannot look more generally at how social and other factors relate to educational attainment and UKCAT performance in the entire set of medical school applicants (rather than entrants). The analysis also considers only simple predictor-outcome correlations, and makes no attempt to calculate construct validity, taking into account the unreliability of predictor and outcome measures, restriction of range due to selection, and the right-censorship of predictor variables such as A-level scores. All of that is considered in a separate paper, which carries out a meta-regression of construct validity not only in the UKCAT-12 study, but also in five other cohort studies [35].

## Methods

The primary dataset for the UKCAT-12 study consists of the 4,811 students in three separate cohorts, who entered medical school in 2007, 2008 or 2009, and for whom outcome measures were available at the end of their first academic year. For those cohorts, UKCAT was used in selection by 23, 25 and 26 medical schools. The secondary datasets contained data on a range of other measures, including prior educational achievement, socioeconomic background, and so on. Many of the secondary measures are missing, either for structural reasons (for example, some socio-economic measures are only available for England; A-levels were not available for mature entrants; and so on) and others were also sporadically missing, probably mostly at random. Some secondary measures do not describe individual students, but instead are **contextual variables**, describing not the students themselves, but features of the educational and socioeconomic environment in which the students lived prior to joining medical school (for example, aggregated measures of the attainment of the secondary school attended, socio-economic measures of the local community where the student lived, and so on). Contextual measures need to be treated with care, but have been included not only for their sociological interest, but also because similar measures are now provided routinely by UCAS, and have been shown to relate to achievement at the BMAT aptitude test [36]. Table 1 summarizes the many measures which were in the analysis, and more detailed information can also be found in the UKCAT-12 Technical Report [34]. The measures can be divided into six broad categories.

**Table 1:** Summary of variables in the analysis

**Category**	**Variables used in the analysis**	**Notes and comments**
Medical school outcome data	**Outcome first year 4 pt**	Medical school data on student performance in their first academic year for the three cohorts. Not all schools provided data for all cohorts - 11, 11 and 9 schools providing data for the 2007 to 2009 cohorts, for 1,661, 1,710 and 1,440 students. In the same cohorts, UKCAT was used for selection by 23, 25 and 26 medical schools. The overall number of students from the 12 schools varied from 87 to 945 (median = 335, mean = 401, SD = 243). Medical schools were asked to provide several items of information on each student, although not all schools provided all information. Data were collected by the UKCAT Consortium Office, and not by the researchers. Measures used were as follows: **OutcomeFirstYear4pt** : Outcome of the first year on a four-point scale (Passed all exams at first attempt; passed after re-sitting exams; repeating the first year; and leaving the course); **OverallMark**, **TheoryMark**, and **SkillsMark**: Averaged percentage marks in medical school assessments. **OverallMark**, based on all assessments, was available for 4,510 students, one school providing only **OutcomeFirstYear4pt**, and occasional students elsewhere not having percentage marks; in each case a proxy **OverallMark** was calculated as a normal score, using SPSS’s Rank Cases/Normal Scores command. Separate marks were also available for ‘Theory’ and ‘Skills’ assessments, the definition of theory and skills being left to medical schools. **TheoryMark** and **SkillsMark** were available for 2,075 and 3,184 students. Because percentage marks are not necessarily comparable across schools, **OverallMark**, **TheoryMark** and **SkillsMark** were standardized to a mean of zero and SD of one within medical schools and cohorts.
	**Overall mark**	
	**Theory mark**	
	**Skills mark**	
Prior educational achievement	**Alevel_number_total**	We will describe the analysis of A-levels in some detail. Other examinations show minor variations from the analysis of A-levels which we will then describe.
	**Alevel_number_total Alevel_Totalbest**	
	**Alevel_TotalPoints**	*A (Advanced) levels.* Scored as A = 10, B = 8, C = 6, D = 4, E = 2, Else = 0. A* grades at A level were not awarded during the study period. Measures were only calculated for students with three or more A-levels, others being set as missing. Fourteen measures separate measures were obtained, described further in the Technical Report [34]. General Studies was not counted in the overall totals, means and so on, but was analyzed separately, as its status is unclear. The measures (with their names in bold), were: **Alevel_number_total**: Number of non-General Studies A-levels, of the 2,764 entrants, 41.8% had 4 or more; **Alevel_Totalbest**: Sum of the three highest A-level grades, which was 73.0% of students, was the maximum score of 30 (that is, AAA), with 21.3% scoring 28 (AAB), 5.0% scoring 26 (ABB/AAC), 0.6% scoring 24 (BBB or equivalent), and four candidates scoring 20, 16, 16 and 10. **Alevel_TotalPoints**: Total points achieved by a student for all of A-levels, which for those taking three A-levels was the same as the previous measure; **Alevels_Taken_1_or_more_Biology**, **Alevels_Taken_1_or_more_Chemistry**, **Alevels_Taken_1_or_more_Physics**, and **Alevels_Taken_1_or_more_Maths**): a series of ‘dummy variables’, scored as 1 if the subject had been taken and 0 if it had not. 95.7%, 99.1%, 24.8% and 63.3% of A level students had A levels in Biology, Chemistry, Physics and Math. **Alevels_highest_Biology**, **Alevels_highest_Chemistry**, **Alevels_highest_Physics**, and **Alevels_highest_Maths**: Highest grade attained by a student on Biology, Chemistry, Physics and Math subjects; except for Math, students mostly had taken only one exam in each category; **Alevels_Taken_1_or_more_NonScience** was a 1/0 dummy variable indicating that a student had A-level(s) other than in the core sciences of Biology, Chemistry, Physics or Math (or General Studies). A total of 49.9% of the students had at least one non-science A-level; **Alevels_Taken_1_or_more_GeneralStudies**: A 1/0 dummy variable indicating whether a student had taken General Studies A-level; 26.0% had done so; **Alevels_highest_GeneralStudies**: For students taking General Studies, the highest grade attained, 46.9% having an A grade;
	**Alevels_Taken_1_or_more_Biology**	
	**Alevels_Taken_1_or_more_Chemistry**	
	**Alevels_Taken_1_or_more_Physics**	
	**Alevels_Taken_1_or_more_Maths**	
	**Alevels_highest_Biology**	
	**Alevels_highest_Chemistry**	
	**Alevels_highest_Physics**	
	**Alevels_highest_Maths**	
	**Alevels_Taken_1_or_more_NonScience**	
	**Alevels_Taken_1_or_more_GeneralStudies**	
	**Alevels_highest_GeneralStusdie**	
	In addition equivalent variables for other qualifications are named in similar ways but with **Alevel…** replaced by **ASlevel…**, **GCSE…**, **SQAhigher…**, **SQAhigherPlus…** and **SQAadvHigherPlus…** .	
	**EducationalAttainmentGCE**	
	**EducationalAttainmentSQA**	
	**EducationalAttainment**	
	**zEducationalAttainmentGCE**	
	**zEducationalAttainmentSQA’**	
	**SQAorGCE**	
		*AS (Advanced Subsidiary) levels.* Variables are similar to those for A-levels except that they are named **ASlevel…** rather than **ALevel…** Scored as for A levels (A = 10, B = 8, C = 6, D = 4, E = 2, Else = 0). Measures are similar except that students had to have taken at least four AS-levels, and totals were for the best four AS-levels achieved. For reasons which are not clear, fewer students had 4+ AS-levels (n = 1,877) than had 3+ A-levels (n = 2,764). AS-level grades showed more variability than A-levels, only 56.3% of students scoring a maximum 40 points for their best grades, compared with 73.0% of students gaining 30 points from their best three A-levels.
		*GCSE (General Certificate of Education)*. Variables are broadly similar to those for A-levels except that they are named **GCSE…** rather than **ALevel…** GCSE results were only available for the 2009 entry cohort. Single subjects were scored as A* = 6, A = 5, B = 4, C = 3, D = 2, E = 1, else = 0 and double Science and other subjects were scored as A*A* = 12, A*A = 11, and so on, and counted as two GCSEs taken. Very few students had eight or fewer GCSEs, and therefore overall scores were therefore based on the nine best grades. GCSE scores were available for 930 students, and were more variable than A-levels or AS-levels, only 16.6% of students having the maximum of 54 points (equivalent to 9 A* GCSEs). Scores were calculated for the four individual core sciences, and score were also calculated for Combined Science (taken by 32.8% of students). **GCSE_Number_NonScience_Exams**: Because all students had taken several non-science subjects, this variable was the number of non-science subjects taken.
		*Scottish Highers.* Measures are broadly similar to those for A-levels, except that names begin **SQAhigher…** Grades were scored as A = 10, B = 8, C = 6 and D = 4. Students were only included who had five or more grades at Highers, the five highest being summed. Other differences from A-levels are that there is no General Studies component, and almost all students will take a non-science Higher. Results for Scottish Highers were available for 769 students, 72.4% gaining a maximum score of 50 points based on best five grades.
		*‘Scottish Highers Plus’*. This is a construction of our own, reflecting the fact that although Scottish Highers are scored by UCAS and by most Scottish universities as A, B, C and D, the UCAS grades are actually A1, A2, B3, B4, C5, C6 and D7. These results, with two bands at each grade, are treated as meaningful by many English universities (although not, it would seem, Scottish universities), and therefore we also scored Highers on a basis of A1 = 10, A2 = 9, B3 = 8, B4 = 7, C5 = 6, C6 = 5 and D7 = 4. We have named this as ‘Scottish Highers Plus’, and variable names begin **SQAhigherPlus…** . These results have a wider range of scores, only 19.9% of students gaining the maximum 50 points.
		*Scottish Advanced Highers.* Variable names begin **SQAadvHigherPlus…** . Many Scottish universities seem not to require Advanced Highers, an argument against their use being that only selective schools have the resources or provide the possibility of studying Advanced Highers, and hence there are concerns about widening access. We note, however, that in this group of students, of 478 applying from the state sector, 93.1% had one or more Advanced Highers, compared with 81.8% of 237 nonstate sector entrants. Overall, 573 students in the present survey had at least two Advanced Highers (that is, 74.5% of the 769 students with Highers), and a further 108 had one Advanced Higher. We, therefore, also assessed the predictive value of Advanced Highers. Scoring was as for “Scottish Highers Plus” (that is, A1 = 10, A2 = 9, B3 = 8, B4 = 7, C5 = 6, C6 = 5 and D7 = 4), with scores calculated for individual core science subjects, along with highest overall score attained. 22% of the 694 students with at least one Advanced Higher had a maximum of 10 points on their best Advanced Higher, and 22.6% had 7 or fewer points.
		*Overall measures of educational attainment.* As described in the text, an overall measure of educational attainment was calculated for each student, **EducationalAttainmentGCE** or **EducationalAttainmentSQA** for GCE and SQA assessments. These variables were based on a set of eight or ten measures respectively, with missing values replaced by the EM algorithm, and then the first principle component extracted. A single variable, **EducationalAttainment** was created which was the z score of either **EducationalAttainmentGCE** or **EducationalAttainmentSQA,** whichever was not missing**.** Because the present analysis is interested in measures within medical schools, **EducationalAttainmentGCE** and **EducationalAttainmentSQA** were also standardized to have a mean of zero and SD of one within each medical school cohort, to produce the variables **zEducationalAttainmentGCE** and **zEducationalAttainmentSQA**. We also used a dummy variable, **SQAorGCE**, to indicate whether entrants had taken Scottish or other qualifications. Note that in the paper on Construct Validity [35] the unstandardized measures were used, in order that information on applicants as well as entrants could be on a common scale.
UKCAT measures	**zUKCATtotal**	Data were provided by the UKCAT consortium, with some additional measures calculated by HIC in Dundee. The overall measure of performance was the total score, **UKCATtotal**, and there were also scores on the four subscales **UKCATabstractReasoning**, **UKCATdecisionAnalysis**, **UKCATquantitativeReasoning**, and **UKCATverbalReasoning**. Each of the measures was also standardised as a z-score within medical schools and cohorts, to give **zUKCATtotal**, with the four subscales being **zUKCATabstractReasoning**, **zUKCATdecisionAnalysis**, **zUKCATquantitativeReasoning**, and **zUKCATverbalReasoning**, There was also information on the date of taking UKCAT, the variable **UKCATdayOfTakingPctileRank** giving relative date of taking the test within cohorts, low scores indicating early takers of the test. Not all candidates answered all questions, in most cases probably because they ran out of time, and as a result on average had lower scores than if they had guessed at items, the measure **UKCATskipped** giving the overall number of skipped items, which had a median of 4, only 25.9% of candidates answering all items. Some candidates were allowed extra time because of special needs, which is indicated by the variable **UKCATexamSeriesCode**; on average these candidates had higher overall scores than other candidates.
	**zUKCATabstractReasoning**	
	**zUKCATdecisionAnalysis**	
	**zUKCATquantitativeReasoning**	
	**zUKCATverbalReasoning**	
	**UKCATskipped**	
	**UKCATdayOfTakingPctileRank**	
	**UKCATexamSeriesCode**	
	**UKCATcandPerSchool**	
		In their analyses of BMAT [36], Emery *et al.* reported that candidates from schools with more extensive experience of the test performed somewhat differently, and therefore a contextual variable, **UKCATcandPerSchool**, was provided by HIC which counted the number of candidates taking UKCAT in a student’s school since the test’s inception.
Schooling measures	**SelectiveSchool**	Some information on schooling, including school codes, was available from UCAS, and the school codes could also be linked into contextual data available from the Department for Education (DfE; formerly DFES) at Key Stage 5 for the academic year 2010 (file created May 2011), for schools in England. The merging of the two datasets was carried out by HIC. School type was available from two separate sources, UCAS and DFES. In UCAS’s data, of 4,811 students, 69 had missing information, 360 were in UCAS’s ‘Unknown’ category, 219 were ‘Apply Online UK’, and 86 were ‘Other’. Of 4,077 students for whom information was available, 1,941 (47.6%) were classified as coming from Selective Schools (‘Grammar School’ or ‘Independent School’), and 2,136 (52.4%) from non-Selective Schools (‘Comprehensive School’, ‘Further/Higher education’, ‘Sixth Form Centre’ and ‘Sixth Form College’). The DFES database also had a measure of Selective Schooling, with information on 2,830 individuals available, of whom 1,387 (49.0%) attended selective schools. The overlap of the UCAS and DFES classifications was good, but not perfect. Our final measure, entitled **SelectiveSchool** had a value of 1 if either UCAS or DFES data suggested a school was selective, and otherwise was 0. Altogether of the 4,811 individuals in the Primary Database, information was available for one or both sources in 4,114 cases, of whom 1,986 (48.3%) had evidence of having attended a selective school.
	**DFESshrunkVA**	
	**DFES.AvePointStudent**	
	**DFES.AvePointScore:**	
		*Contextual school measures*. The DfE data had a total of 22 contextual measures on schools. After a range of preliminary, exploratory analyses we confined the analyses to three variables: **DFESshrunkVA**, which is a measure of value added between Key stages 4 and 5, and was available for the schools of 2,561 students; **DFES.AvePointStudent**, which is a measure of the average points gained by each student at a school across all of that school’s examination entries, and was available for the schools of 2,586 students; and **DFES.AvePointScore**, which is a similar measure to the previous one except that the average is at the level of examination entries (rather than students), and was available for the schools of 2,582 students.
Demographic measures	**UK**	*Nationality* was based on the online information provided when students took UKCAT; of 4,811 students, 4,598 (95.6%) were UK nationals, 176 (3.7%) were EU/EEA nationals and 37 (0.8%) were from outside the EU/EEA; the binary variable was called **UK**.
	**UCAS.male**	
	**CAND.Age**	
	**CAND.AgeGT21**	*Sex* was based on information provided by UCAS; of 4,811 students, 2,081 (43.3%) were male and 2,730 (56.7%) were female. The variable was called **UCAS.male**, scoring 1 = male and 0 = female.
	**CAND.Age30plus**	
	**UCAS.Ethnic2.**	*Age* was based on stated age in years when taking the UKCAT test, and ranged from 17 to 45 (mode = 18, mean = 19.55, SD = 2.84). Age was missing in 45 cases, 28.9% of students were aged 21+, and 1.3% were aged 30+. The variable was called **CAND.Age**. Additional 0/1 variables were created to indicate whether candidates were 21 or older or 30 or older (**CAND.AgeGT21**, **CAND.Age30plus**).
		*Ethnicity* was based on the standard 23 categories in the UCAS coding. Ethnicity was missing in 69 cases, for 214 was coded as Unknown, and for 192 was coded as ‘Not given’. On a simplified six category basis there were 3,057 White, 577 Indian sub-continent, 223 Other Asian, 92 Black, 140 Mixed and 60 Other. For simplicity, and as in many other studies [37]) we grouped students as White (n = 3,057, 73.7%) and Non-White (n = 1,092, 26.3%), in a variable called **UCAS.Ethnic2**.
Socio-economic measures	**CAND.NSSEC**	*Socio-economic classification (SEC)*, variable **CAND.NSSEC**, was based on the online information provided by students taking UKCAT, who completed the abbreviated version of the self-coded questionnaire (NS-SEC) provided by UK National Statistics2. SEC was calculated separately for each parent (if provided), and the higher SEC used. Of 4,091 individuals with usable information, 3,740 (91.4%) were in SEC group 1, 105 (2.6%) in group 2, 146 (3.6%) in group 3, 38 (0.9%) in group 4, and 62 (1.5%) in group 5, where group 1 has the highest status.
	**IMDOverallQualityDecile**	
	**IMD1IncomeDecile**(with two subscales)	
	**IMD2EmploymentDecile**	
	**IMD3HealthDisabilitySkillsDecile**	
	**IMD4EducationDecile** (with two subscales),	*Socio-economic contextual measures.* For applicants living in England, postcodes for place of residence were used to link to small-area census statistics collected as part of The English Indices of Deprivation [38] and which generate a series of Indices of Multiple Deprivation (IMD). For ease of analysis, HIC converted the measures to deciles, low scores indicating greater deprivation. **IMDOverallQualityDecile** provides an overall single indicator of deprivation. In addition there are 15 more detailed scales and subscales, whose names are moderately self-explanatory: **IMD1IncomeDecile** (with two subscales), **IMD2EmploymentDecile**, **IMD3HealthDisabilitySkillsDecile**, **IMD4EducationDecile** (with two subscales), **IMD5HousingAndServicesDecile** (with two subscales), **IMD6CrimeDecile**, **and IMD7LivingEnvironmentDecile** (with two sub-scales). Note that although these scales are described in terms of deprivation, they are scored as 1 = high deprivation and 10 = low deprivation, and therefore are renamed as ‘Quality’ so that higher scores indicate a higher quality on the measure.
	**IMD5HousingAndServicesDecile** (with two subscales)	
	**IMD6CrimeDecile,**	
	**IMD7LivingEnvironmentDecile** (with two sub-scales).	

Variables are indicated by their SPSS variable names (in bold) to reduce ambiguity.

1. MEDICAL SCHOOL OUTCOME DATA. Medical schools provided information on overall outcome on a four-point scale (passed all exams at first attempt; passed after re-sitting exams; repeating the first year; and leaving the course), which we called **OutcomeFirstYear4pt.** Average percentage marks on assessments were also available for most students (**OverallMark**), and for many students separate marks were also available for ‘theory’ and ‘skills’ assessments (**TheoryMark** and **SkillsMark**; see Table 1). The overall, theory and skills marks were all based on marks attained at the first attempt.

2. PRIOR EDUCATIONAL ATTAINMENT, AND SO ON. Information on prior educational attainment was provided by UCAS, consisting of Scottish Higher and Advanced Higher results for students from Scotland (collectively SQA qualifications), and A-level, AS-level and GCSE results for other students (collectively GCE qualifications). Educational qualifications are always complex to analyze, because different candidates take different examinations with different structures and grading schemes, and candidates have chosen to study different subjects. Four medical schools were from Scotland and eight from the rest of the UK, entrants to the former mostly, but not entirely, taking Scottish Highers rather than A-levels. No easy solution is possible for the difficult problem of equating the two different sets of results [37], and we have followed the approach of Tiffin *et al.*[19] in converting Scottish Highers and A-levels to z-scores, which can then be combined. The Technical Report [34] describes an extensive set of preliminary analyses of the wide range of different measures of attainment (see Table 1). Briefly, each of the 42 derived scores for A-levels, AS-levels and GCSEs was correlated with **OverallScore** (TR Table 1^a,b^). Multiple regressions suggested that only a subset of eight measures (TR Table 2) showed independent predictions of outcome. Missing values for these eight measures were replaced by expectation-minimization (EM) imputation, the resulting 8 x 8 correlation matrix factor analyzed, the first principle component extracted, (which has a mean of zero and SD of one), and scores on that were used as an optimal summary measure of attainment at A-level, AS-level and GCSE. A similar process was carried out for the 51 derived measures of Highers, ‘Highers Plus’ and Advanced Highers, each of which was correlated with **OverallScore** (TR Table 3). Ten independent predictors were found, missing values replaced by imputation, and the first principle component extracted (TR Table 4). Since the principle components for the two analyses were both on standardized scales, they could be combined to provide an optimal summary measure of **Educational Attainment** for the majority of students. Educational attainment measures differ between medical schools and between cohorts, but because the main interest in this study is prediction within medical schools, we have standardized **Educational Attainment** within cohorts and medical schools, resulting in the variable we call **zEducational Attainment**. It should be noted that educational qualifications were only available in most cases for non-mature students (age less than 21). Although statistically optimal, and hence good for assessing underlying processes using as much information as possible, we realize that **zEducational Attainment** does not reflect the current selection processes, and therefore we also report results for the more conventional measures of three best A-levels, four best AS-levels, nine best GCSEs, five best Scottish Highers, five best Scottish “Highers Plus” (which includes finer definition of bands within grades), and the best Scottish Advanced Higher.

3. UKCAT SCORES. The main measures from UKCAT were the scores on the cognitive tests, the total score, **UKCATtotal**, and the scores on the four subtests, **UKCATabstractReasoning, UKCATdecisionAnalysis, UKCATquantitativeReasoning** and **UKCATverbalReasoning**. Details of the tests can be found elsewhere [20,38-42]. Formats were unchanged across the three cohorts. Reliabilities are summarized in the Technical Report [34] (p.18). Mean UKCAT scores differed both between medical schools, and scores also rose across the cohorts, the differences being meaningful since UKCAT is statistically equated across cohorts using item-response theory. As with educational attainment, UKCAT scores have, therefore, been standardized as z-scores within medical schools and cohorts, since it is performance within medical school and cohort which is of interest. As well as scores on UKCAT, we also had measures of the date of taking the test **(UKCATdayOfTakingPctileRank)**, the number of items not answered **(UKCATskipped)**, whether there was a time extension because of special needs **(UKCATexamSeriesCode)**, and the contextual measure of the experience of a student’s secondary school in taking UKCAT **(UKCATcandPerSchool)**.

4. SCHOOLING MEASURES. The principal measure was **SelectiveSchool**, which used data from UCAS and DFES to identify candidates educated at selective secondary schools. Three contextual measures were also used, **DFESshrunkVA, DFES.AvePointStudent,** and **DFES.AvePointScore**, which assessed the performance of students at the secondary school attended by the student in our study (see Table 1 for details).

5. DEMOGRAPHIC MEASURES. Measures were available of **Nationality** (UK or non-UK), **Sex, Age** and **Ethnicity** (classified for present purposes as White/non-White). See Table 1.

6. SOCIO-ECONOMIC MEASURES. Socio-economic classification (SEC) was based on the online information provided by students taking UKCAT, who completed the abbreviated self-coded questionnaire (NS-SEC) of UK National Statistics [43]. Postcode based contextual measures of social background were based on the 16 measures provided in The English Indices of Deprivation [44] (see Table 1).

**Table 2:** Correlations of UKCAT sub-scores with outcomes

	**Abstract**	**Decision**	**Quantitative**	**Verbal**	**OverallMark**	**SkillsMark**	**TheoryMark**
	**reasoning**	**analysis**	**reasoning**	**reasoning**			
Abstract reasoning **(zUKCATabstractReasoning)**	1	.196***	.190***	.114***	.080***	.053**	.052*
		(4,811)	(4,811)	(4,811)	(4,811)	(3,184)	(2,075)
Decision analysis **(zUKCATdecisionAnalysis)**	.196***	1	.156***	.146***	.090***	.056***	.077***
	(4,811)		(4,811)	(4,811)	(4,811)	(3,184)	(2,075)
Quantitative reasoning **(zUKCATquantitativeReasoning)**	.190***	.156***	1	.213***	.076***	.044*	.079***
	(4,811)	(4,811)		(4,811)	(4,811)	(3,184)	(2,075)
Verbal reasoning **(zUKCATverbalReasoning)**	.114***	.146***	.213***	1	.115***	.028	.177***
	(4,811)	(4,811)	(4,811)		(4,811)	(3,184)	(2,075)
Total UKCAT score **(zUKCATtotal)**	.604***	.655***	.583***	.591***	.148***	.075***	.160***
	(4,811)	(4,811)	(4,811)	(4,811)	(4,811)	(3,184)	(2,075)
Educational attainment **(zEducationalAttainment)**	.144***	.131***	.133***	.087***	.362***	.210***	.351***
	(3,432)	(3,432)	(3,432)	(3,432)	(3,432)	(2,240)	(1,407)
Three best A-levels **(Alevels_TotalBest)**	.123***	.121***	.127***	.062**	.177***	.096***	.248***
	(2,764)	(2,764)	(2,764)	(2,764)	(2,764)	(2,000)	(1,250)
Five best highers **(SQAhighers_TotalBest)**	.083*	.129***	.202***	.070	.003	.027	.074
	(769)	(769)	(769)	(769)	(769)	(298)	(199)

Correlations of the UKCAT subscales with each other, with UKCAT total score, and with prior Educational Attainment (three best A-levels, five best Highers and **zEducationalAttainment)**, and medical school performance (Overall, and Skills and Theory assessments); ***P <.001; **P <.01; *P <.05.

**Table 3:** Simple Pearson correlations of key measures with a range of demographic, school, social and UKCAT process measures

		**Educational Attainment**	**3/5 best A-levels/Highers**	**UKCAT total score**	**Overall medical school**
		**(zEducational Attainment)**	**(Alevels_TotalBest**	**(zUKCATtotal)**	**score (OverallScore)**
			**SQAhighers_TotalBest)**		
Demographic measures	UK national **(UK)**	.008	.000/-.042	.060 ***	-.007
		(3,432)	(2,764/769)	**(4,811)**	(4,811)
	Male **(UCAS.Male)**	-.037 *	.026/.058	.061 ***	-.039 **
		**(3,432)**	(2,764/769)	**(4,742)**	**(4,742)**
	Aged 21+ **(Cand.AgeGT21)**	n/a	n/a	-.060 ***	.080 ***
				**(4,766)**	**(4,766)**
	Aged 30+ **(Cand.Age30plus)**	n/a	n/a	-.023	-.003
				(4,766)	(4,766)
	Ethnic2 (non-White) **(UCAS.Ethnic2)**	-.053 **	-.062 **/-.033	-.141 ***	-.142 ***
		**(3,221)**	**(2,549/**766**)**	**(4,149)**	**(4,149)**
School measures	Selective schooling **(SelectiveSchool)**	.051 **	.038 */.120 ***	.075 ***	-.101 ***
		**(3,432)**	**(2,764/769)**	**(4,811)**	**(4,811)**
	DfES value added KS 5 **(DFESshrunkVA)**	-.012	.012/n/a	-.014	-.049 *
		(2,092)	(2,119)	(2,561)	**(2,561)**
	DfES average points per student **(DFES.AVEPointStudent)**	.085 ***	.127 ***/n/a	.097 ***	-.065 ***
		**(2,114)**	**(2,141)**	**(2,586)**	**(2,586)**
	DfES average points per exam entry **(DFES.AvePointScore)**	.111 ***	.101 ***/n/a	.044 *	-.111 ***
		**(2,109)**	**(2,136)**	**(2,582)**	**(2,582)**
Social background	Socio-economic classification (SEC) (1 = High 5 = Low) **(CAND.NSSEC)**	-.058 *	-.084 ***/-.046	-.056 ***	-.011
		**(2,939)**	**(2,356/675)**	**(4,091)**	(4,091)
	Overall deprivation decile (1 = high, 10 = low deprivation) **(IMDOverallQualityDecile)**	.079 ***	.076 ***/n/a	.113 ***	.032
		**(2,275)**	**(2,307)**	**(3,074)**	(3,074)
	Income deprivation decile **(IMD1IncomeDecile)**	.078 ***	.083 ***/n/a	.125 ***	.039 *
		**(2,275)**	**(2,307)**	**(3,074)**	**(3,074)**
	Employment deprivation decile **(IMD2EmploymentDecile)**	.063 **	.073 ***/n/a	.109 ***	.008
		**(2,275)**	**(2,307)**	**(3,074)**	(3,074)
	Health disability decile **(IMD3HealthDisabilitySkillsDecile)**	.055 **	.048 */n/a	.098 ***	.016
		**(2,275)**	**(2,307)**	**(3,074)**	(3,074)
	Education deprivation decile **(IMD4EducationDecile)**	.056 **	.064 **/n/a	.083 ***	-.019
		**(2,275)**	**(2,307)**	**(3,074)**	(3,074)
	Housing and services deprivation decile **(IMD5HousingAndServicesDecile)**	.046 *	**.035/**n/a	-.024	.059 ***
		**(2,275)**	**(2,307)**	(.176)	**(3,074)**
	Crime deprivation decile **(IMD6CrimeDecile)**	.061 **	**.040/**n/a	.100 ***	.062 ***
		**(2,275)**	**(2,307)**	**(3,074)**	**(3,074)**
	Living environment decile **(IMD7LivingEnvironmentDecile)**	.049 *	**.036/**n/a	.066 ***	.042 *
		**(2,275)**	**(2,307)**	**(3,074)**	**(3,074)**
UKCAT measures	UKCAT questions skipped/missed **(UKCATskipped)**	.000	-.015/-.041	-.310 ***	-.005
		(3,432)	(2,764/769)	**(4,811)**	(4,811)
	UKCAT percentile day of taking test **(UKCATdayOfTakingPctileRank)**	-.092 ***	-.059 **/-.018	-.058 ***	-.090 ***
		**(3,432)**	**(2,764**/769**)**	**(4,811)**	**(4,811)**
	UKCAT allowed extra time **(UKCATexamSeriesCode)**	.007	.014/-.012	.030 *	.004
		(3,432)	(2,764/769)	**(4,811)**	(4,811)
	UKCAT school experience of test **(UKCATcandPerSchool)**	-.029	.038/-.174 ***	-.033 *	-.033 *
		(3,295)	**(**2,630**/754)**	**(4,022)**	**(4,022)**
GCE and SQA results	Three best A-levels **(Alevels_TotalBest)**	.690 ***	−/−	.088 ***	.177 ***
		**(2,725)**		**(2,764)**	**(2,764)**
	Four best AS-levels **(ASlevels_TotalBest)**	.605 ***	.416 ***/-	.155 ***	.184 ***
		**(1,842)**	**(1,865/-)**	**(1,877)**	**(1,877)**
	Nine best GCSEs **(GCSEs_TotalBest)**	.600 ***	.293 ***/-	.202 ***	.082 *
		**(721)**	**(723/-)**	**(930)**	**(930)**
	Five best Scottish Highers **(SQAhighers_TotalBest)**	.328 ***	−/−	.040	.003
		**(715)**		(769)	(769)
	Five best Scottish “Highers Plus” **(SQAhighersPlus_TotalBest)**	.532 ***	-/.884 ***	.104 **	.137 ***
		**(682)**	**(−/730)**	**(730)**	**(730)**
	Best Scottish advanced higher **(SQAadvHighers_TotalBest)**	.776 ***	-/.249 ***	.118 **	.362 ***
		**(639)**	**(−/769)**	**(681)**	**(681)**

**Note**: measures in italics are **contextual measures**, and should be treated with care as they describe the student’s environment rather than the student themselves. **Key**: * *P* <.05; ** P <.01; *** *P* <.001. Correlations with *P* <.05 are also shown in bold. Names in bold in parentheses are variable names as described in Table 1).

**Table 4:** Comparison of the four outcome groups

	**Fail (a)**	**Repeat 1st**	**Passed after**	**Passed all**	**ANOVA (r) Linear**	**ANOVA (r)**	**Levene test**	**Homogenous**
		**year (b)**	**re-sits (c)**	**first time (d)**	**trend F(1,n)**	**Nonlinear F(2,n)**		**subsets (s)**
**Overall Mark** (p, q); All cases	**−2.644**	**−1.924**	**−1.110**	**.235**	2843.8	36.9	*P* ≤.001	a,b,c,d
	(1.28, 96)	(.99, 94)	(.79, 565)	(.80, 4,056)	*P* <.001	*P* <.001		
**Theory Mark** (p)	**−1.258**	**−1.322**	**-.654**	**.250**	619.9	23.1	NS	ab, c, d
	(.44, 29)	(.82, 29)	(.76, 294)	(.68, 1,723)	*P* <.001	*P* <.001		
**Skills Mark** (p)	**−1.079**	**-.891**	**-.616**	**.214**	602.8	32.0	*P* ≤.001	ab, bc, d
	(.84,40)	(.87, 438)	(.90, 438)	(.71, 2,655)	*P* <.001	*P* <.001		
Totoal UKCAT score **(UKCATtotal)**	**2492**	**2457**	**2486**	**2544**	43.7	6.8	NS	abc, ad
	(192, 96)	(230,94)	(205, 565)	(205,4,056)	*P* <.001	*P* = .001		
zUKCAT (p) **(zUKCATtotal)**	**-.121**	**-.312**	**-.186**	**.036**	25.3	5.3	NS	abc, ad
	(.95,96)	(1.01, 94)	(.99, 565)	(.99, 4,056)	*P* <.001	*P* = .005		
UKCAT abstract reasoning (p) **(zUKCATabstractReasoning)**	**-.163**	**-.224**	**-.096**	**.022**	13.1	0.75	NS	abcd
	(.94, 96)	(1.02, 94)	(.97, 565)	(1.00, 4,056)	*P* <.001	NS		
UKCAT decision analysis (p) **(zUKCATdecisionAnalysis)**	**-.064**	**-.302**	**-.129**	**.026**	14.01	3.70	NS	abc, ad
	(1.08, 96)	(.99, 94)	(.98, 565)	(.995, 4,056)	*P* <.001	*P* = .025		
UKCAT quantitative reasoning (p) **(zUKCATquantitativeReasoning)**	**.045**	**-.110**	**-.116**	**.018**	3.38	3.38	NS	abcd
	(.97, 96)	(1.03, 94)	(1.06, 565)	(.99, 4,056)	NS	*P* = .034		
UKCAT verbal reasoning (p) **(zUKCATverbalReasoning)**	**-.087**	**-.136**	**-.127**	**.023**	9.56	2.13	NS	abcd
	(.97, 96)	(1.16, 94)	(.96, 565)	(1.00, 4,056)	*P* = .002	NS		
Educational attainment (p) **(zEducationalAttainment)**	**-.441**	**-.653**	**-.563**	**.104**	156.1	29.5	*P* ≤.001	abc, d
	(.942, 65)	(1.06, 60)	(1.14, 414)	(.94, 2,893)	*P* <.001	*P* <.001		
Three best A-levels **(Alevels_TotalBest)**	**28.89**	**28.28**	**29.06**	**29.39**	44.8	6.47	*P* ≤.001	a,bc,d
	(2.82, 56)	(1.58, 49)	(1.36, 333)	(1.22, 2,326)	*P* <.001	*P* = .002		
Five best highers **(SQAhighers_TotalBest)**	**48.71**	**47.50**	**48.70**	**48.99**	3.8	1.66	NS	abcd
	(1.57, 17)	(2.58, 16)	(2.46, 88)	(2.40, 648)	NS	NS		

Notes:

*P*-values are standardized within schools (Note: this is not the case for the raw, UKCAT total mark)

q. In a small proportion of cases, as described in the text, the overall mark is based on a normal score derived from the four-point categorical scale. For comparability with other analyses, the first row includes these cases. However, the second row analyses only cases where an overall mark was explicitly provided.

r. The denominator df, n, can be calculated as N-4, N is the total number of cases (provided in individual cells).

s. If values are together then they are not significantly different from one another and form a homogenous subset with *P* >.05 using the Ryan-Einot-Gabriel-Welsch range test. As an example, “abc, ad” means that groups a, b and c (Fail, repeat first year and passed after re-sits) do not differ from one another; likewise groups a and d (Fail, Passed all first time) do not differ from one another. Group d (Passed all first time) is significantly different from Repeat first year and Passed after re-sits. “a, b, c, d” indicates each differs from each of the other three groups, and “abcd” indicates no significant *post hoc* differences.

### Ethics, anonymity and confidentiality

Ethical permission for the study was provided by UCL. The medical schools providing data for the analysis did so on the basis of strict anonymity of the institutions themselves. We have also had no access to raw, non-anonymized data, and have had to accept the data as provided as being correct and accurate. Data were provided by the Health Informatics Centre (HIC) at the University of Dundee as anonymized, encrypted files, with a randomized identification code for applicants in each year, which allowed merging of various datasets. Data analysis was carried out by ICM and CD. SN and JD did know the identity of medical schools, but did not process the anonymized data.

### Statistical analyses

Conventional statistical analyses used IBM SPSS 20 (International Business Machines Corporation, Statistical Package for the Social Sciences, Armonk, New York, USA), with missing values handled using the EM method in Missing Values Analysis. Multilevel modeling used MLwiN v 2.24. (Centre for Multilevel Modelling, University of Bristol, Bristol, UK).

## Results

The UKCAT-12 study has one set of outcome measures (medical school performance measures), two important sets of predictors (measures of prior educational attainment and scores on the UKCAT test), and a wide range of background measures (demographic, secondary schooling, socio-economic and other measures). These will be considered in turn, and in relation to each other.

### Medical school outcome measures

**Figure 1:**
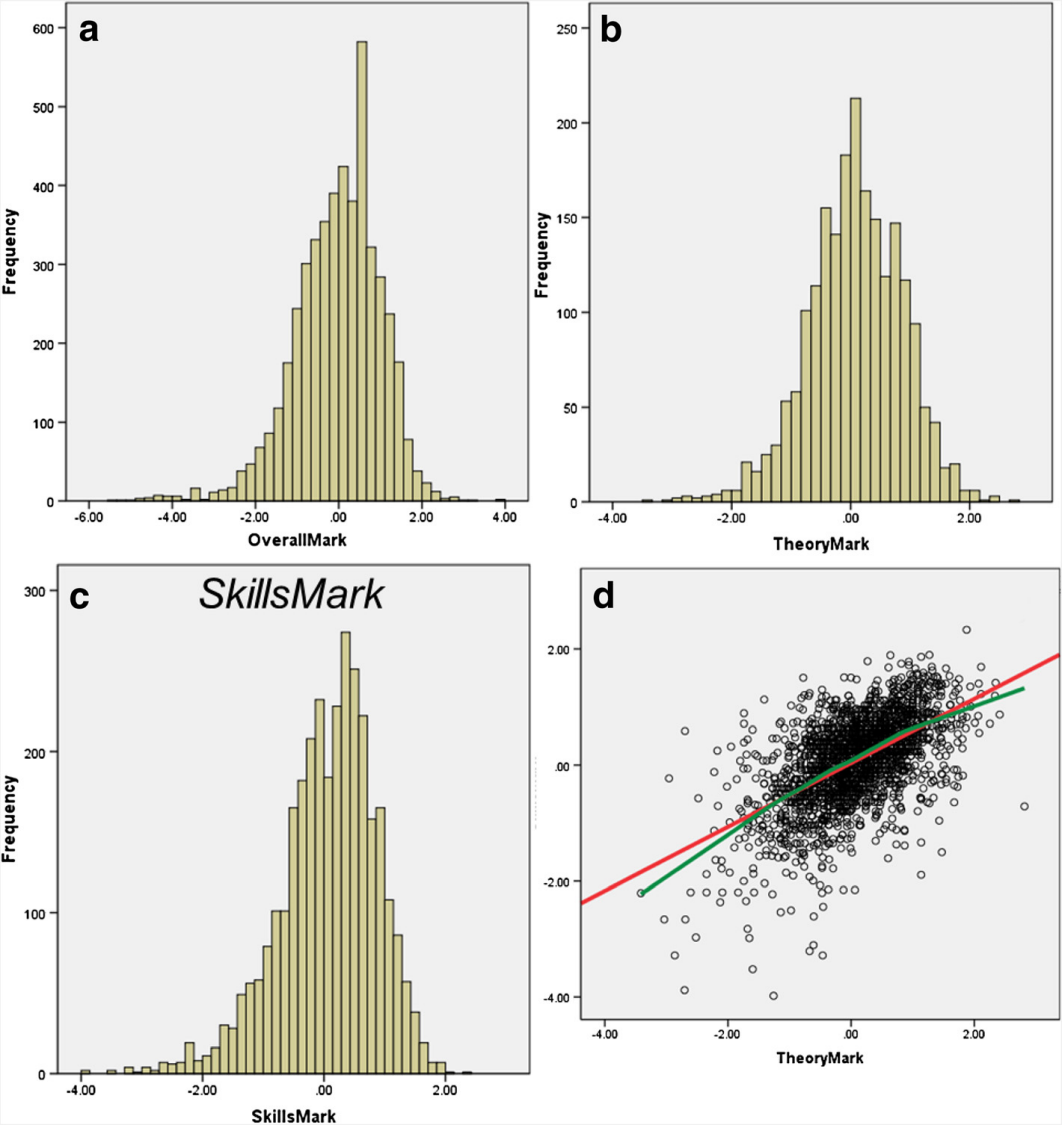
**OverallMark (a), TheoryMark (b) and SkillsMark (c) , and SkillsMark in relation to TheoryMark (d).** The red line is the linear regression, and the green line a lowess curve.

Of 4,811 medical students on the four-point outcome scale **(OutcomeFirstYear4pt)**, 4,056 (84.3%) passed all their first year examinations without re-sits, 565 (11.7%) progressed from the first year after re-sits, 94 (2.0%) were required to repeat the first year, and 96 (2.0%) left the medical school (proportions which are very similar to the 81%, 14%, 1% and 4% reported in a cohort of medical students entering in 1981 [45]. Altogether 109 students left medical school, in 55 cases for Academic Reasons, and in 49 for non-Academic reasons (3 after repeating the first year, and 10 after passing the first year exams). Figure 1a shows that the distribution of **OverallMark** is approximately normal, with some leftward skew. Distributions of **TheoryMark** and **SkillsMark** in Figure 1b,c are also approximately normally distributed, the correlation between them being 0.566 (Figure 1d).

### Background variables and their relationship to educational attainment, UKCAT score and medical school outcome

Table 3 shows correlations of the 22 background variables, as well as the 6 conventional measures of GCE and SQA achievement, with **zEducationalAttainment, zUKCATtotal** score, and performance at medical school. It should be remembered that many of the background variables are themselves inter-correlated, and in the following analyses multivariate statistics are used to tease apart the relationships. Among this population of entrants to medical school, who are not, of course, representative of applicants, the 22 background variables together accounted for 3.9% of variance in educational attainment, and 14.0% of variance in UKCAT total score, although that difference may in part reflect selection on educational attainment and, hence, greater restriction of range.

### Prior educational attainment and its relation to medical school performance

**Figure 2:**
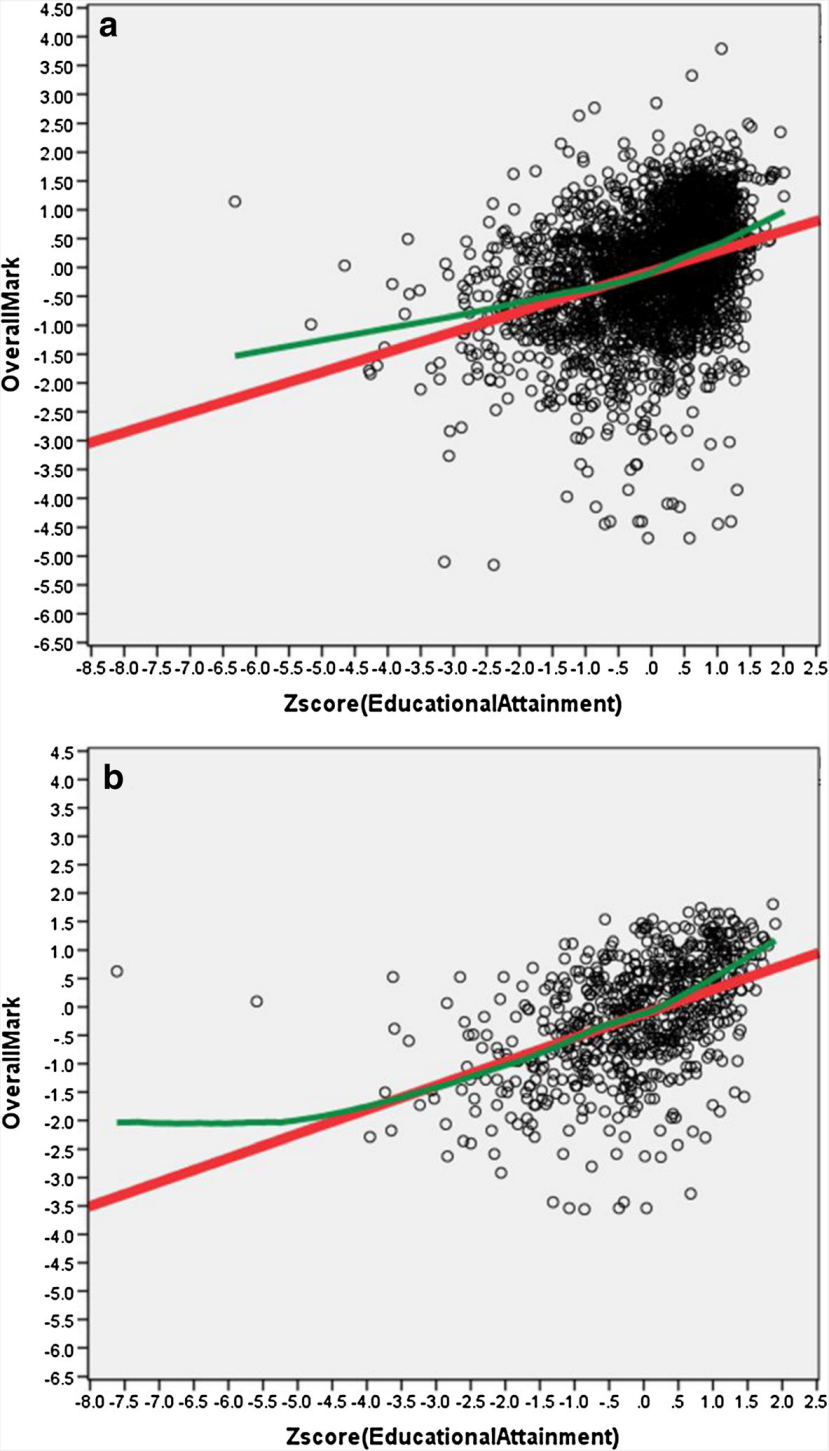
**Relationship of OverallMark at medical school to Educational Attainment (zEducationalAttainment).** Scattergrams are shown separately for **a)** General Certificate of Education (GCE) qualifications (A-levels/AS-levels/GCSEs), and **b)** Scottish Qualifications Authority (SQA) qualifications (Scottish Highers and Advanced Highers). The red line is a linear regression, and the green line is a lowess curve. The slope of the line for SQA qualifications (b = .423) is significantly larger than that for GCE qualifications (b = .349; interaction term, t = 25.95, 3,428 df, *P* <.001).

Overall there was a highly significant correlation between prior educational attainment **(zEducationalAttainment)** and **OverallMark** (r = .362, n = 3432, *P* <.001), which was significantly stronger (z = 3.76, *P* <.001) for SQA qualifications (r = .464, n = 715, *P* <.001, 95% CI .406 to .522) than for GCE qualifications (r = .331, n = 2,717, *P* <.001, 95% CI .298 to .364), the relationships being shown in Figure 2. **OverallMark** was not as strongly correlated with the more conventional measures of three best A-levels (r = .185, n = 2,717, *P* <.001) and five best Scottish Highers (r = .121, n = 715, *P* = .001), primarily due to restriction of range and ceiling effects, although both correlated strongly with **zEducationalAttainment** (A-levels: r = .690, n = 3,432, *P* <.001; Highers; r = .328, n = 715, *P* <.001). Students with lower attainment at A-level did, though, perform less well, the regression model suggesting that students with BBB performed about 1.1 SDs below those with AAA, a substantial effect. As explained in the Technical Report [34], our analysis looked in detail at various aspects of measures of Educational Achievement. In particular, we note that for GCE examinations predicting **OverallMark:** i) AS-level results provided an incremental prediction over A-levels; ii) GCSEs provided an incremental prediction over A- and AS-levels; iii) grade at General Studies A-level provided an incremental prediction over (other) A-levels; iv) grades on all four core-sciences provided an incremental prediction over summed A-level grades, further exploration finding that a key predictor appears to be the minimum core science grade attained, low values predicting poorer performance at medical school; v) there was no evidence that grades at any of the four core sciences were particularly predictive of medical school performance, with simple correlations of **OverallMark** with grades in Biology, Chemistry, Math and Physics being .182, .143, .125 and .172 (all *P* <.001, n = 2,645, 2,739, 18,750 and 685). For SQA examinations in relation to **OverallMark**: i) ‘Highers Plus’ scoring provides incremental prediction over conventional Highers scoring; ii) Advanced Highers provides incremental prediction over Highers/HighersPlus; iii) None of the core sciences showed specific incremental prediction, either at Highers or HighersPlus; iv) Advanced Highers grades at Biology and Chemistry (but not Math and Physics), provided incremental prediction; v) As with A-levels, the minimum core science grade attained seems to have predictive value. Finally, because SQA qualifications had a higher predictive validity than GCE qualifications, we compared the predictive validity of the qualifications in Scottish medical schools (where entrants have either GCE or SQA qualifications) and other medical schools (where entrants have GCE qualifications). In Scottish medical schools, SQA results had higher correlations with outcome than did GCE results for students on the same course, whereas GCE predicted outcome equivalently in Scottish and non-Scottish schools. SQA results do have greater predictive power, perhaps because of the inclusion of Advanced Highers results. However, elsewhere we show that despite the higher correlation with outcome, the construct validity of SQA results is somewhat lower than that for GCE results [35].

### Predictive value of background variables, after taking prior educational attainment into account

**Figure 3:**
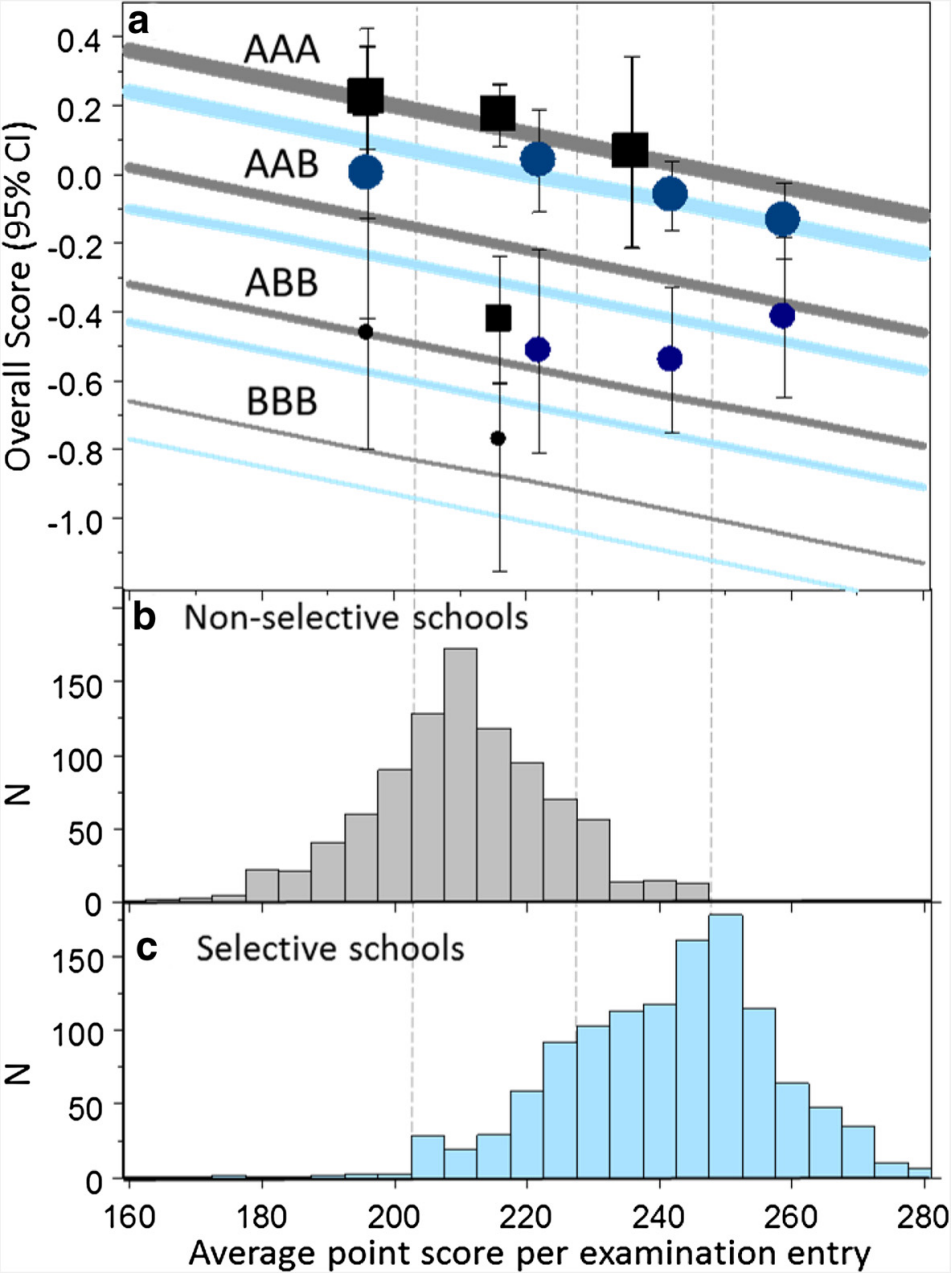
**a) Performance at medical school in relation to DfES average point score for secondary school attended.** Performance of medical school entrants (vertical) is expressed as a standardised (z) score. DfES measure of average point score per examination entry (horizontal) is for the (English) secondary school which the entrant attended. Sections **b)** and **c)** show the distribution of average point scores for entrants from non-selective secondary schools (, in gray) and selective secondary schools **(c**, in blue**)**. The gray and blue lines in **a)** show the fitted regression lines for non-selective secondary schools (gray) and selective secondary schools (blue), for candidates with AAA at A-level (top, thickest line), down through AAB and ABB to BBB (lowest, thinnest line). Average point scores are grouped into four groups, indicated by vertical dashed lines, and mean entry scores, with 95% CI, are shown for entrants from non-selective secondary schools (black squares) and selective secondary schools (blue circles), the largest squares/circles for AAA, the medium squares/circles for AAB, and the smallest squares/circles for ABB. Groups with small N and, hence, large CIs are omitted.

Prior educational attainment correlates with a wide range of background variables (TR Table 5). An important question, though, concerns the extent to which background variables continue to predict outcome after educational attainment has been taken into account. **OverallMark** was regressed on the 22 background variables, with an alpha set at 0.001 to account for repeated testing. Four background measures were significant, in order of entry: **Ethnic2**, non-White students performing less well (beta = −.126, *P* <10^-14^); **being a mature student,** students over the age of 21 performing better than non-mature students (beta = .057, *P* <.001); **UKCATdayOfTakingPctileRank**, students who took UKCAT late performing less well (beta = −.089, *P* <10^-7^); and **DFES.AvePointEntry**, students from high-attaining secondary schools performing less well (beta =−.085, *P* <10^-7^). Figure 3 explores **DFES.AvePointEntry** in more detail. Figure 3c shows that average points per exam entry are substantially lower in non-selective schools than selective schools. The average points are divided into four groups (boundaries 205, 230 and 250), with almost no selective secondary schools in the lowest group and almost no non-selective secondary schools in the highest group. Effects upon overall score were estimated with a regression model in which there were significant effects of three best A-level grades (beta = .205, *P* <.001) and secondary school-level average points (beta = −.085, *P* = .005), and an almost significant effect of selective secondary schooling (beta = −.056, *P* = .059), the fitted regression lines being shown in Figure 3a. There was no evidence of interactions. Actual mean scores are shown for candidates whose secondary schools were in the four groups of average points, and it can be seen, particularly for entrants with AAA grades in both non-selective and selective secondary schools that overall scores at medical school are lower in those from secondary schools with higher average point scores. From the regression lines it can be estimated that one grade at A-level (the difference between AAA and AAB) is equivalent to 85 points on the average point score, so that an entrant with ABB at A-level from a secondary school with an average score of 175 (at about the 1st percentile of the non-selective schools) performs similarly at medical school to a candidate with AAA at A-level from a secondary school with an average score of 265 (at the 99th percentile of the selective secondary schools).

### The relationship of UKCAT total scores to medical school performance and to background variables

**Figure 4:**
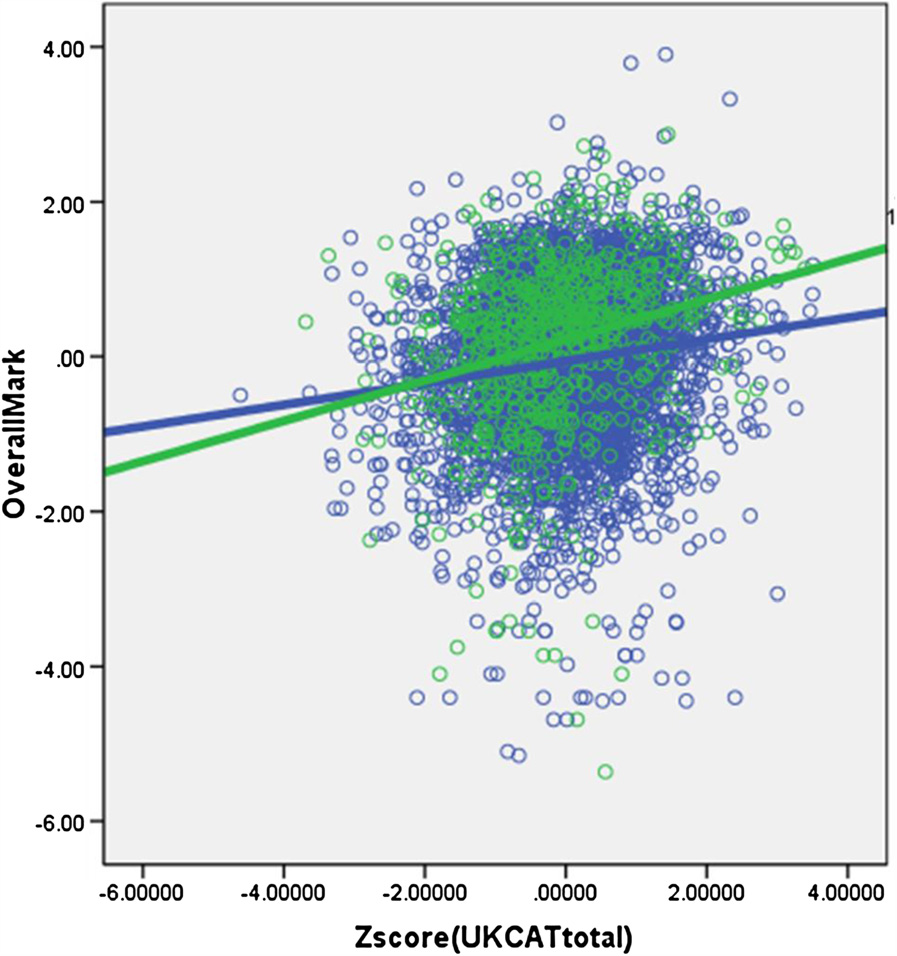
**Scattergram showing relationship between OverallMark at medical school, and UKCAT score (standardised within medical schools).** Mature students (green) and non-mature students (blue) are shown separately, along with fitted linear regression functions. The crossing of the two lines is at about 2.5 standard deviations below the mean, so that at almost all candidate ability levels, mature students outperform non-mature students, with a steeper slope for mature students.

The correlation between UKCAT scores and **OverallMark** was 0.148 (n = 4,811, *P* <0.001). Because UKCAT is often said to be particularly helpful in selecting mature entrants (where educational qualifications are often out of date or not applicable), we compared the predictive validity of UKCAT in mature and non-mature students (Figure 4). The correlation with **OverallMark** was higher in mature students (r = .252, N = 690, *P* <.001), than in non-mature students (r = .137, n = 4,076, *P* <.001). Mature students had somewhat more variable raw UKCAT scores (SD = 237.7 compared with 200.0), but regression showed that that was not the cause of the increased correlation with **OverallMark**. The incremental validity of UKCAT after taking educational attainment into account was assessed by regressing **OverallMark** firstly on **zEducationalAttainment**, and then on **zUKCATtotal. zUKCATtotal** significantly improved the prediction of **OverallMark** (t = 3.54, 3,429 df, *P* <.001), but the beta coefficient was only 0.057, whereas the beta coefficient for **zEducationalAttainment** after taking UKCAT into account was 0.351. In practice, many admissions tutors can only use three best A-levels and, therefore, we repeated the exercise with **Alevel_TotalbestN**, when the beta for UKCAT was .101 but for A-levels was .168. The previous analysis of **OverallMark** in relation to the background variables, after taking educational achievement into account, had found four background variables related to medical school performance. We repeated the regression analysis after inclusion of UKCAT as well as educational achievement. All of the four variables significant previously were again significant, suggesting that UKCAT performance cannot eliminate the effects of ethnicity, secondary schooling, day of taking UKCAT, or being a mature student in first year medical school performance. In addition, **Sex** was also a significant predictor of **OverallMark**, males performing less well, after taking UKCAT and other measures into account (beta = −.056, *P* <.001).

### Theory and Skills exams and the subtests of UKCAT

UKCAT has four subtests - Abstract Reasoning, Decision Analysis, Quantitative reasoning and Verbal Reasoning - which may correlate differently with educational attainment and with medical school performance, particularly perhaps between Theory and Skills measures. Table 2 shows correlations between the four UKCAT sub-scales, and it can be seen that they are significantly, but only moderately, correlated, suggesting that they are indeed measuring different cognitive processes. Each sub-scale correlates with the total UKCAT score (but it is, of course, a part of it). The subscales all correlate to much the same extent with educational attainment, except for verbal reasoning which has a rather lower correlation. All four sub-scales correlate with **OverallMark** at medical school, although verbal reasoning correlates significantly more highly than the other three sub-scales, a pattern which is clearer still for the marks from Theory exams, whereas all four sub-scales show low and similar correlations with **SkillsMark**. In the Technical Report [34], we describe further analyses showing that of the subtests, it is Verbal Reasoning, which particularly contributes unique variance to predicting medical school performance after Educational Achievement has been taken into account, both overall and for **TheoryMark**, and also for **SkillsMark**, when higher Verbal Reasoning predicted a lower **SkillsMark**. Verbal ability may predict better than other subtests due to verbal tests being less subject to practice and coaching effects [26].

### Identifying students in the four outcome groups

Although medical school performance is a continuous measure, students eventually end up in one of four categories, **OutcomeFirstYear4pt**, with the lower categories having important consequences for the students and their careers. We, therefore, compared the four groups on UKCAT scores and the measures of prior educational attainment. Table 4 shows comparison between the groups using one-way analysis of variance. The overall pattern is that the students who perform less well tend to score lower on the various measures. An exception is that in several cases the group who had failed showed scores that were higher than those who are repeating the first year, as for instance, on measures of prior educational attainment and on several of the UKCAT scores. That may be because the group of failures is not homogenous. Numbers in the Fail group are relatively small, with 55 leaving for academic reasons and 49 for non-academic reasons (although reasons for leaving medical school are often complex and not readily classified [46]). A comparison is available in the Technical Report [34].

### Differences between medical schools assessed using multilevel modeling

Medical schools differ [47], and it is possible that variables which predict outcome in one medical school will predict better or worse in other medical schools. The 12 medical schools in UKCAT-12 allow such possibilities to be assessed. The analyses will begin with a model of the importance of educational achievement, which will be described in some detail, and then a number of other factors will be considered as well.

### Prior educational achievement

**Figure 5:**
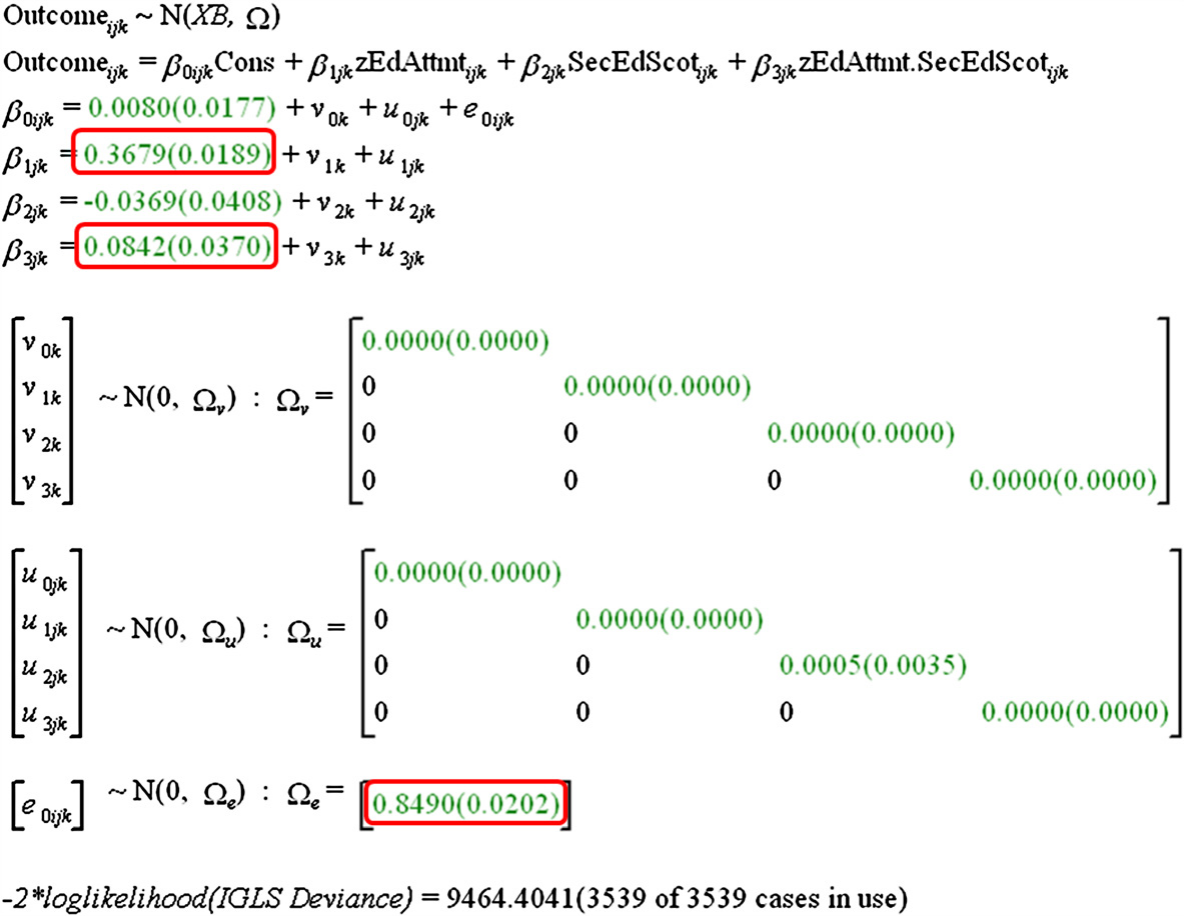
**Multilevel modeling of relationship of OverallMark at medical school to Educational Attainment (zEducationalAttainment).** See text for details.

A three-level model is fitted (see Figure 5), with individual students at the first level, who are nested within the 12 medical schools at the second level, which in turn at the third level are nested within either Scotland or elsewhere. Note that because **zEducationalAttainment** is not available for mature students, this analysis is restricted to non-mature students. The outcome variable is **OverallMark**, which is standardized within medical schools and cohorts (and hence overall effects typically have means close to zero). The main predictor is **zEducationalAttainment**. A dummy variable, at the student level, **SQAorGCE** indicates whether students took SQA or GCE qualifications (and it has already been suggested that SQA attainment predicts medical school outcome better than GCE attainment). **OverallMark** can be predicted at level-1 by **zEducationalAttainment** and **SQAorGCE**, and by their interaction. The slope of the regression of **OverallMark** on **zEducationalAttainment** and **SQAorGCE**, and their interaction can also show variance between medical schools and between Scottish and non-Scottish medical schools. Figure 5 shows the full fitted model, estimates being shown with their standard errors in parentheses, so that estimates are significantly different from zero with *P* <.05 on a two-tailed test if their value is at least twice their standard error (and these are indicated in Figure 5). **zEducationalAttainment** is a strong indicator of **OverallMark**, the interaction with SQA qualifications is also significant, predicting **OverallMark** more strongly than do GCE qualifications. No other terms are significant, which in particular means a) that educational achievement has the same predictive value in all 12 medical schools, irrespective of whether they are in Scotland or elsewhere, and b) the increased predictive effect of SQA qualifications is the same in all medical schools, in Scotland or elsewhere. The important conclusion is that although medical schools might have differed in the predictive value of educational attainment, perhaps because of differences in teaching methods, curriculum or whatever, there is no evidence that they do so.

### Sex, secondary schooling and ethnicity

The three-level model for **zEducationalAttainment** was fitted with the addition of sex, the contextual secondary school attainment measure **DFES.AvePointEntry**, and ethnicity (White), as well as the interactions of those measures with **zEducationalAttainment**. After taking **zEducationalAttainment** into account, male students underperformed (estimate = −.0699, SE .0309), non-White students underperformed (estimate = −.2504, SE = .0357), and students from secondary schools with a higher **DFES.AvePointEntry**, score (that is, higher-attaining secondary schools) performed less well overall (estimate = −.1112, SE = .0183). There was no evidence that any of the measures interacted with educational attainment and particularly important for interpreting these results is that there was no evidence, for any of the three measures, of variance between the 12 medical schools. In other words, males, for instance, underperformed to the same extent in all 12 medical schools, which is important for understanding and interpreting such effects.

### UKCAT scores and age

**Figure 6:**
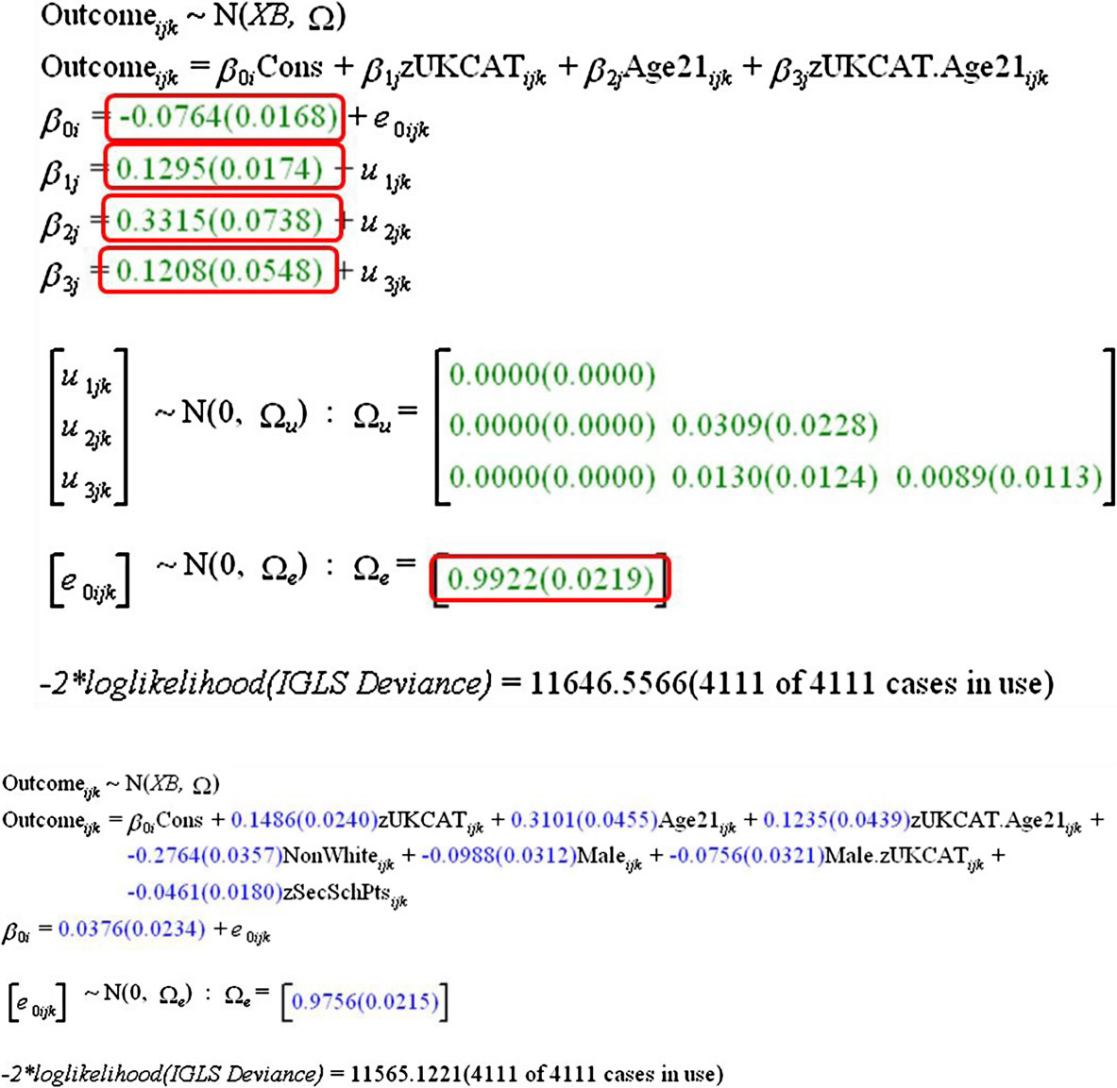
**Multilevel modeling of relationship of OverallMark at medical school to UKCAT total score.** See text for details.

Multilevel modeling of the prediction of **UKCATtotal**, as well as the subscores, was broadly similar to that for educational attainment, except that differences between medical schools in Scotland and elsewhere were not considered, so that the model had two levels. Age of students (<21, 21+) was included as a previous analysis suggested that UKCAT predicted better in mature students. The overall fitted model is shown in Figure 6. UKCAT significantly predicts outcome (estimate = .1295, SE .0174). Mature students also perform better than non-mature students (.3315, SE .0738), and there is a significant interaction between maturity and UKCAT, UKCAT predicting more strongly in mature students (estimate = .1208 SE .0548). There was no evidence that UKCAT, age or the interaction of UKCAT and age behaved differently in their predictive ability at any of the 12 medical schools.

### UKCAT and sex, secondary schooling and ethnicity

As before, sex, the contextual secondary school attainment measure **DFES.AvePointEntry**, and ethnicity (White) were added into the model, as also were their interactions with UKCAT. Level-2 effects for each term were also considered, but none were significant, indicating that the measures behaved similarly in all of the 12 medical schools. Level 2 effects were, therefore, removed from the model. At level 1, and after taking UKCAT into account and with educational attainment, there was underperformance by male students (−.0927 SE .0379), non-White students (−.2888 SE .0371), and those from high achieving secondary schools (−.0493, SE .0184). The only significant interaction with UKCAT was for male sex (−.0756 SE .0321), UKCAT having a stronger prediction of outcome in female students than male students.

## Discussion

The primary focus of UKCAT-12, the first large-scale, collaborative UK study involving 12 medical schools, was to assess the predictive validity of the UKCAT test of aptitude for medical school. A key strength is the large sample size of nearly 5,000 medical students which provides adequate statistical power for answering questions that a single medical school or a single cohort could not, as well as providing answers that are likely to generalize to other medical schools. In addition, the collecting of a wide range of other measures of educational attainment, socio-economic background, and education contextual measures, as well as outcome data, from 12 medical schools means that a much wider range of issues could be addressed.

Inevitably, UKCAT-12 has some limitations. It was intentionally restricted for this paper to first-year examination results, in part to make the analyses manageable, and in part because the end of the first year is a time when it is particularly common to leave medical school. Although our outcome measures do not (as yet) have measures of performance as doctors, there can at least be confidence that those leaving the medical school will not make good doctors, for indeed they will not become doctors at all. Other analyses make clear that performance across different years of undergraduate and postgraduate performance shows high stability, resulting in what we have called the ‘Academic Backbone’ [48], with first year medical school performance strongly predicting subsequent performance. Likewise, exam results before, during and after medical school are correlated, both at the individual level [1,2], and at the medical school level [48,49]. It will, of course, be important and of great interest in the future to extend the current studies into later years, particularly in the clinical years and beyond, in order to assess empirically the extent of prediction.

Although we had separate measures of ‘Theory’ and ‘Skills’ exams, these measures were not available for all students, and for ‘Skills’ measures in particular, a range of different types of assessment was included. In the future a particular interest will be in clinical skills, which as yet it is too soon to measure in all of these cohorts. Nevertheless, we expect that abilities acquired during the early years of medical training, including an understanding of basic medical sciences, will underpin the understanding of clinical science, so that it is highly likely that those underperforming in their first year will on aggregate also underperform on measures of clinical ability.

UKCAT-12 only included 12 medical schools, but there is reason to believe they are broadly representative of UK medical schools, with the possible exception that a small group of the most selectively intense and academic of medical schools, particularly including Oxford and Cambridge, choose instead to use BioMedical Admissions Test (BMAT). Since many medical school candidates necessarily end up taking both UKCAT and BMAT, a collaborative research exercise involving both aptitude tests might usefully illuminate the extent of similarities and differences between them. On the basis of the very limited published evidence for the validity of BMAT [21,23,50], which was in a single medical school, it seems probable that its predictive validity is of a similar order of magnitude to UKCAT. Likewise, it is probable that the results generalize to other selection tests which are primarily of intellectual aptitude.

There are many important and useful findings from the UCKAT-12 study, and here we will briefly overview some of them, starting with more general conclusions about selection and performance overall, and then considering UKCAT in particular.

### Prior educational attainment

Academic qualifications, typically A-levels and Scottish Highers, have long been the mainstay of medical student selection, the course being academically very demanding, and an ability to cope both with the cognitive load and the science content being seen as important. UKCAT-12 confirms the importance of prior educational attainment and performance on the course correlating with educational attainment. The role of A-levels and Highers has been questioned, in large part because there is little variation in educational attainment in students actually on the course. However, UKCAT-12’s large samples make clear that even small amounts of under-attainment (AAB, ABB or BBB compared with AAA at A-level) correlate with poorer performance in the first year of medical school. There is, therefore, clear evidence justifying not only the continued use of school achievement for selection but also supporting the development of validated approaches utilizing more of the available information (such as GCSE, AS-level and additional details, such as ‘Highers Plus’ and Advanced Highers). A key question for selection concerns widening access and two aspects merit consideration here. First, the analysis of construct validity, described in detail in a separate paper [35], allows the calculation of how well those with lower qualifications are likely to perform if admitted. Second, we can now make justifiable estimates of how contextual measures, such as personal and educational background might be ‘factored in’, in such a way that they optimize the potential of medical school entrants best.

### Secondary school achievement

The UKCAT-12 identified a number of aspects of educational achievement of practical importance.

1. Although most medical schools require a qualification in Chemistry, there are few data asking whether Chemistry is a good predictor of outcome, or whether other academic subjects are particularly important. UCKAT-12 found that no particular core science seemed at A-level or Highers to be especially predictive of outcome. Instead, the lowest grade attained in a core science did seem to predict outcome.

2. Grade at A-level general studies showed an independent predictive value, after other A-levels were taken into account.

3. AS-level grades added additional predictive value after taking A-levels into account.

4. GCSE grades added further incremental validity on top of AS- and A-levels. This differs from the recent government claim that GCSEs have equal prediction of degree class as A-levels [51], perhaps because the non-linear relationship is steeper at high attainment levels.

5. There was no evidence that students with ‘Double Science’ at GCSE underperformed [34].

6. Scottish Highers did not predict, mainly due to ceiling effects, although using the full range of marks in Scottish Highers (our ‘Highers Plus’ scores) provided better prediction, and Scottish Advanced Highers produced further prediction.

7. Overall SQA qualifications predicted performance better overall than GCE qualifications (although they appear to have lower construct validity [35].

Educational attainment is clearly a strong predictor of outcome, but it is currently limited by so many applicants getting top grades (a problem which might be partly mitigated by A* grades at A-level, although at present government policy is in the future to decouple AS- and A-level assessments). A partial solution is to consider also AS-levels and GCSE results, both of which have incremental value over A-levels (and a wider range of performance). AS-levels and GCSEs also have the practical advantage of being available at the time of selection, rather than merely being estimated grades for exams yet to be taken. Scottish Highers particularly have the problem of many candidates being at ceiling, but using the full range of marks available at Highers (A1, A2 and so on), as well as using Advanced Highers can result in much better prediction.

Our constructed measure of Prior Educational Achievement demonstrates that more can be achieved by the detailed assessment of secondary school-based performance measures. Though care would need to be taken to assess impact on widening access and this measure cannot be applied to graduate applicants.

In summary, even with the limitations of currently available measures of educational attainment, the results of this study suggest that it would be possible to obtain better prediction of medical school performance by drawing upon information currently neglected in selection: namely AS-level, GCSE, and the full range of marks provided by Scottish Highers, with the introduction of A* grades at A-level also perhaps being of help.

The replicability of these findings, and the reasons for them, can be left for future studies, but all show the potential importance of large-scale studies of selection both for forcing theoretical interpretation on the nature of medical education, and for providing a clear evidence-base for answering important practical questions about selection.

### Ethnicity, sex and age as predictors of medical school performance

The UKCAT-12 data show that older students, female students and white students all perform better at medical school, with all effects being significant after taking educational attainment and UKCAT scores into account. The ethnic difference found is similar to that found in a meta-analysis [52]. The UKCAT-12 study particularly adds to previous studies because the multilevel modeling shows that the effects of maturity, sex and ethnicity are equivalent at all 12 medical schools, making it unlikely that the differentials found in performance are the result of institutionally-specific effects.

### Secondary schooling and socio-economic factors at the individual and the contextual level

Students educated at selective schools performed less well at medical school than those educated in non-selective schools. Contextual measures were also available for secondary schools in England, and students also performed less well who came from secondary schools with a higher value-added score at key stage 5, or at which students gained more points at A-level (with two different ways of calculating those points). Multiple regression showed that the effect was not due to selective secondary schooling as such, but principally related to the average A-level attainment level of pupils at a secondary school, with the apparently paradoxical finding that the higher the achievement of pupils overall, the less well pupils from that secondary school did at medical school. The result is not novel, and a similar finding had previously been reported by the Higher Education Statistics Agency (HESA) [3,53]. The mechanism of the finding is not completely clear, but one possibility is that higher achieving secondary schools achieve their results in part by contributing extra ‘polish’ to a student’s work, polish which no longer generalizes or transfers when they then move to a university where such support is not present. Alternatively, it may be that academic aspirations are higher in those from less academic environments, due to the ‘Big Fish Little Pond Effect’ [54]. That the effect found by HESA is now found in medical students suggests that there is a strong argument for using the contextual measure of average A-level attainment at a secondary school in making admission decisions.

UKCAT-12 also considered a range of other socio-economic measures, both directly of the socio-economic classification of a student themself, based on self-description of parental occupation using the self-completion scale of the Office for National Statistics [43], and indirectly, as contextual measures of deprivation on a range of scales, from the English Deprivation Indices [44]. The findings are straightforward; as has been reported before [45], socio-economic background seems to bear little or no relationship to medical school performance once educational attainment is taken into account. Both in the UK [55] and the US [56], for university admissions in general, evidence suggests that the barrier for college entry is lower application rates, rather than universities discriminating against those from lower socio-economic status (SES) backgrounds. The implication is that socio-economic contextual factors may not be of utility in selection, although they could well be of use in encouraging higher rates of application from disadvantaged groups.

### Predictive validity of UKCAT

Performance at UKCAT does correlate with first-year performance at medical school. The correlation is small but significant for secondary school leavers and is larger for mature entrants. The incremental validity of UKCAT after taking the current educational attainment used for selection into account is small but significant and as such provides sufficient added value for UKCAT to be an adjunct to current selection processes. However, the data analysis also demonstrates the potential of the improved predictive validity of using fuller information on educational attainment. Measures of educational attainment probably predict university outcome better because they provide evidence simultaneously in three domains: intellectual ability (typically fluid intelligence), substantive subject knowledge about science (crystallized intelligence), and a combination of motivation, appropriate study skills and personality [57]. UKCAT aims to assess fluid intelligence and other attributes thought to be important in decision making, but it specifically attempts not to measure science knowledge. The most likely explanation of the relatively lower predictive validity of the UKCAT aptitude tests is that they do not assess domain knowledge (and, hence, indirectly personality, motivation and so on), although personality and motivation are included in more recent versions of UKCAT, they are not included here.

UKCAT predicts outcome significantly better in mature than non-mature students. Mature students often have unusual combinations of academic qualifications, sometimes taken a while ago, and UKCAT is potentially a useful tool for assessing such applicants. UKCAT also predicts differently in males and females, the predictive validity being less in males than females, reflecting in part the fact that males do less well at medical school but do somewhat better overall on UKCAT.

### Improvements to UKCAT

Options for improving the predictive validity of the cognitive tests in UKCAT are several-fold. Reliability of UKCAT could be somewhat improved by increasing the test length, although the relatively low construct validity, described elsewhere [35], suggests that the benefit would not be large. This would also incur increased costs and additional candidate inconvenience. The UKCAT Verbal Reasoning sub-test has the highest correlations with outcome and review of content and style of this sub-test is underway, but without making the test unbalanced, and there is little theoretical sense in, say, downgrading quantitative abilities for a technical, science-based course. Other cognitive subtests might be added, but the ubiquity of ‘g’ probably means that there are no obvious domains of general mental ability which are not currently covered. The performance of Section 2 of BMAT [23] and the science knowledge tests of MCAT [32] suggest that tests which include substantive science knowledge have higher predictive validity than ‘pure’ aptitude tests, which essentially measure only fluid intelligence, and do not assess acquired knowledge or the motivation and personality necessary to acquire it. However, UKCAT originally set out specifically not to include such measures [20] as they are currently available through traditional measures of educational attainment. Non-cognitive measures, primarily of aspects of personality have been piloted by UKCAT, and might contribute additional variance [29]. UKCAT is also currently piloting a situational judgment test, which might also assess separate constructs from those presently assessed.

### Operational utility of UKCAT

The emergence of aptitude testing for medical selection resulted from weaknesses in the existing information available to selectors and these persist. Differentiating in a transparent and fair way between the many applicants who apply for each place in a medical school is challenging and the predictive validity and small incremental validity provide some justification for use of UKCAT. Certainly UKCAT, which is now an integral component of many medical school’s systems, seems in many ways more justifiable than the use of UCAS personal statements, which have been less well studied, are open to criticism for difficulty in scoring consistently, and are subject to a range of influences, including social opportunity, and have not been shown to predict success in medical school [17,58,59]. There is also evidence that utilizing UKCAT has a positive impact on widening access [19]. With on-going refinements and, in particular, the introduction of non-cognitive tools, such as situational judgment tests, the existing assessment may be able to improve further.

### Future directions

The primary impetus for the UKCAT-12 study was to evaluate UKCAT in the context of medical student selection. The data analysis confirms that UKCAT scores have operational utility when used alongside measures of educational attainment. The UKCAT-12 study, we believe, particularly indicates the value of ‘big data’ in evaluating medical education and lessons learned in the UK are probably generalizable elsewhere. A noteworthy feature is the use not only of data collected on students themselves but also integration of other large-scale databases, such as the Department for Education data on secondary school performance in England, and the English Deprivation Indices. For the first time it is possible to consider the construct validity of selection tools for medicine in the UK and these, using the UCKAT-12 study, as well as five other cohort studies, are published separately [35]. Two future developments would be particularly welcome. Though considerable data are now being amassed, it does not cover all worthwhile outcomes, or even all UKCAT medical schools, let alone UK medical schools. Extending studies such as this to include more medical schools with follow-up into later undergraduate years, as well as post-graduate (as has been carried out for other cohort studies [48]), is vital.

In addition, as cohorts of students graduate and go into practice, other databases will become available for addressing questions of predictive validity both for postgraduate assessments and also outcome measures in clinical practice itself, thus enabling a range of large-scale longitudinal studies. Such databases, studies and analyses would enable much faster progress towards the target of the Royal Commission on Medical Education, the Todd Report of 1968, which it called, “an objective evaluation of student selection” [60].

## Conclusions

This collaborative study shows the power of large-scale, multi-medical school, studies of medical education for answering previously unanswerable but important questions about medical student selection, education and training. UKCAT has predictive validity as a predictor of medical school outcome, particularly in mature applicants to medical school. UKCAT offers small but significant incremental validity which is valuable at an operational level where medical schools are currently making selection decisions based on incomplete measures of educational attainment. This study confirms the validity of using all the existing measures of educational attainment in full at the time of selection decision-making. Contextual measures provide little additional predictive value, with the exception of overall level of secondary school attainment, students from high attaining secondary schools performing less well than those from less well attaining secondary schools, as HESA has previously shown for universities in general.

## Endnotes

^a^TR Table 1 refers to “Technical Report Table 1”.

^b^http://www.ons.gov.uk/ons/guide-method/classifications/current-standard-classifications/soc2010/soc2010-volume-3-ns-sec--rebased-on-soc2010--user-manual/index.html

## Abbreviations

A-level: Advanced level; AS-level: Advanced subsidiary level; BMAT: Biomedical admissions test; EM: Expectation-maximization algorithm; GCE: General certificate of education; GCSE: General certificate of secondary education; HESA: Higher education statistics agency; MLwiN: Multi-level modeling for windows; NS-SEC: National statistics socio-economic classification; SEC: Socio-economic classification; SQA: Scottish qualifications authority; UCAS: Universities and Colleges admissions service; UKCAT: United Kingdom clinical aptitude test.

## Competing interests

ICM’s university has received grants from the UKCAT Board during the conduct of the study, and he has on occasion provided advice to UKCAT. CD has received personal fees from the UKCAT Board during the conduct of the study. SN is chair of the UKCAT Board, has sat on the UKCAT research working group during the time of this study, and has not received any personal financial reward or assistance with this study. JSD reports that the University of Dundee is funded by UKCAT to manage and host one of the databases on which the part of this study was based; he has acted as a Board Member of the UKCAT consortium since 2008 and as lead of the UKCAT Research Panel since 2009.

## Authors’ contributions

ICM and CD were commissioned by the UKCAT Consortium to analyze the UKCAT-12 data, and their institutions received a small amount of funding to support the work. The design of the study was a collaboration among all of the authors. ICM and CD carried out the main data analyses, and results were discussed among all participants in the production of the Technical Report. ICM wrote the first draft of the manuscript, and all authors reviewed the final manuscript and contributed to it.
